# 

**Published:** 1906-05-19

**Authors:** 


### Medical Appointments and Vacancies.

### VACANCIES.

Army Medical Service.—Examination of Candidates for not less than Forty Commissions.

Birkenhead Borough Hospital.—Junior Resident House Surgeon.

Birkenhead Union Infirmary, Workhouse, and Sanatorium. Resident Assistant Medical Officer.

Birmingham and Midland Hospital for Skin and Urinary Diseases, John Bright Street, Birmingham.—Clinical Assistant.

Brighton, Sussex County Hospital.—Honorary Assistant Physicians.

East London Hospital for Children and Dispensary for Women, Shadwell, E.—Dental Surgeon.

Evelina Hospital for Sick Children, Southwark.—House Physician and House Surgeon. Also Assistant House Surgeon.

Fareham, Hants County Asylum.—Second Assistant Medical Officer.

Gateforth, near Selby, Leeds Tuberculosis Association Sanatorium.—Resident Medical Officer.

Glasgow Samaritan Hospital for Women.—House Surgeon.

Great Northern Central Hospital, Holloway, N.—Surgeon to the Throat and Ear Department.

Hospital for Women, Soho Square, W.—Surgeon Dentist.

London Hospital, Whitechapel, E.—Surgical Registrar.

New Hospital for Women.—House Physician, Resident Medical Officer, Senior Obstetric Assistant Anæsthetist, and Clinical Assistant.

North-Eastern Hospital for Children, Hackney Road, Bethnal Green, E.—House Surgeon.

North-West London Hospital, Kentish Town Road.—Resident Medical Officer and Assistant Resident Medical Officer.

Royal Westminster Ophthalmic Hospital, West Strand, W.C. Clinical Assistants. Also House Surgeon.

St. Mary's Hospital, Paddington, W.—Surgeon.

Sheffield Children's Hospital (East End Branch).—House Surgeon.

Sheffield Royal Hospital.—Assistant House Physician, unmarried.

Winwick, Warrington, Lancashire County Asylum.—Locum Tenens Medical Officer.

The Chief Inspector of Factories, Home Office, S.W., gives notice of a vacancy as Certifying Surgeon under the Factory and Workshop Act at Cavendish, in the county of Suffolk.

A Drawing-room Meeting will be held at Mrs. Scott's, 11 Kensington Court, W., in aid of the Infants' Hospital, on May 18, at 3 p.m., when the Mayor of Huddersfield will preside. Dr. Saleeby will speak on "Infant Mortality."

### THE BURDETT SERIES

### OF POPULAR TEXT-BOOKS ON NURSING.

**Each Volume of this Series contains about 100 pages written by a competent authority on the subject treated.**

Bound in cloth boards. Price **1**/- each post free; or the Eleven Volumes complete **10**/- post free.

No. 1.—**PRACTICAL HINTS ON DISTRICT NURSING.** By Miss Amy Hughes.

No. 2.—**HOSPITAL AND PRIVATE NURSING.** By Miss S. E. Orme.

No. 3.—**THE MIDWIVES' POCKET-BOOK.** With Prefatory Chapter on the Midwives Bill. By Miss Honnor Morten, Author of "The Nurse's Dictionary," &c. &c.

No. 4.—**FEVERS AND INFECTIOUS DISEASES:** their Nursing and Practical Management. By William Harding, M.D.Ed., M.R.C.P.Lond., Author of "Mental Nursing," &c.

No. 5.—**NOTES ON PHARMACY AND DISPENSING FOR NURSES.** By C. J. S. Thompson.

No. 7.—**THE CARE OF CONSUMPTIVES.** By W. H. Daw, M.R.C.S., L.R.C.P. Lond.

No. 8.—**HOME NURSING OF SICK CHILDREN.** By J. D. E. Mortimer, M.B., F.R.C.S., L.S.A.

No. 9.—**MENTAL NURSING.** By William Harding, M.D.Ed. M.R.C.P.Lond.

No. 10.—**SICK NURSING AT HOME.** By Miss L. G. Moberly.

No. 11.—**GYNÆCOLOGICAL NURSING.** By G. A. Hawkins-Ambler, F.R.C.S., Author of "The Commonwealth of the Body."

No. 12.—**SYLLABUS of LECTURES to NURSES.** By Andrew Davidson, M.D.

***Obtainable of all Booksellers, or of***

**THE SCIENTIFIC PRESS, LTD.,**

**Nursing Publishers and Booksellers,**

**28 & 29 SOUTHAMPTON STREET, STRAND, LONDON, W.C.**

### "THE UNIFORM SYSTEM OF ACCOUNTS."

By SIR HENRY BURDETT, K.C.B.

### THE INDEX OF CLASSIFICATION

Whereby every item of expenditure may be dealt with under identical heads by every group of Institution is now published separately, price 1s.; 1s. 1d. post free.

THE SCIENTIFIC PRESS, LTD.,

28 and 29 Southampton Street, Strand, London, W.C.

